# A novel methylation signature predicts extreme long-term survival in glioblastoma

**DOI:** 10.1007/s11060-024-04741-z

**Published:** 2024-06-19

**Authors:** Brecht Decraene, Grégoire Coppens, Lien Spans, Lien Solie, Raf Sciot, Isabelle Vanden Bempt, Frederik De Smet, Steven De Vleeschouwer

**Affiliations:** 1 Department of Neurosciences, Experimental Neurosurgery and Neuroanatomy Research Group, Leuven Brain Institute (LBI), KU Leuven, Leuven, Belgium; 2 Department of Neurosurgery, University Hospitals Leuven, Leuven, Belgium; 3 Laboratory for Precision Cancer Medicine, Translational Cell and Tissue Research Unit, KU Leuven, Leuven, Belgium; 4 The Leuven Institute for Single-Cell Omics (LISCO), KU Leuven, Leuven, Belgium; 5 Clinical Division and Laboratory of Intensive Care Medicine, Department of Cellular and Molecular Medicine, KU Leuven, Leuven, Belgium; 6 Department of Human Genetics, University Hospitals Leuven, KU Leuven, Leuven, Belgium; 7 Department of Pathology, University Hospitals Leuven, KU Leuven, Leuven, Belgium

**Keywords:** Glioblastoma, Long-term survival, Epigenetics, DNA-methylation

## Abstract

**Purpose:**

Glioblastoma (GBM) is the most common malignant primary brain tumor with a dismal prognosis of less than 2 years under maximal therapy. Despite the poor prognosis, small fractions of GBM patients seem to have a markedly longer survival than the vast majority of patients. Recently discovered intertumoral heterogeneity is thought to be responsible for this peculiarity, although the exact underlying mechanisms remain largely unknown. Here, we investigated the epigenetic contribution to survival.

**Methods:**

GBM treatment-naïve samples from 53 patients, consisting of 12 extremely long-term survivors (eLTS) patients and 41 median-term survivors (MTS) patients, were collected for DNA methylation analysis. 865 859 CpG sites were examined and processed for detection of differentially methylated CpG positions (DMP) and regions (DMR) between both survival groups. Gene Ontology (GO) and pathway functional annotations were used to identify associated biological processes. Verification of these findings was done using The Cancer Genome Atlas (TCGA) database.

**Results:**

We identified 67 DMPs and 5 DMRs that were associated with genes and pathways - namely reduced interferon beta signaling, in MAPK signaling and in NTRK signaling - which play a role in survival in GBM.

**Conclusion:**

In conclusion, baseline DNA methylation differences already present in treatment-naïve GBM samples are part of genes and pathways that play a role in the survival of these tumor types and therefore may explain part of the intrinsic heterogeneity that determines prognosis in GBM patients.

**Supplementary Information:**

The online version contains supplementary material available at 10.1007/s11060-024-04741-z.

## Introduction

Glioblastoma (GBM) remains the most common primary malignant brain tumor with a median survival of 15 months [1]. Although histologically well defined, recent insights show that GBM is an umbrella term that conceals a biologically heterogeneous group of tumors with differences in tumor behavior and consequently survival. The precise phenomena underlying the biological variations remain unclear to date, these are of paramount importance in deciphering potential prognostic and possibly even therapeutic markers. Here, we aim to further elucidate the epigenetic contribution to tumor heterogeneity by comparing the DNA methylation pattern in a unique subset of GBMs from extremely long-term survivors (eLTS) with those from median-term survivors (MTS). By investigating the DNA methylation landscape of this unique GBM subset and comparing it with the landscape of 41 MTS GBM, we identified several robust differences in methylation pattern between the two survival groups.

## Materials and methods

### Patient cohort

**Fig. 1:**
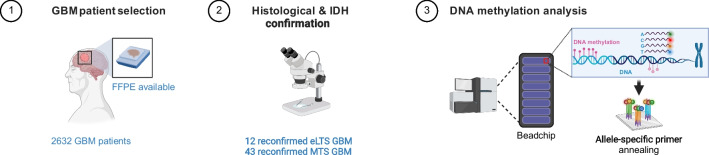
Flow chart depicting the selection of GBM patients with FFPE material available, further diagnostic reconfirmation and final DNA methylation analysis

Records of 2632 high-grade glioma patients who had a therapeutic contact at our tertiary institute (University Hospitals Leuven; UHL) between 2000–2019 were comprehensively reviewed for the diagnosis of GBM (Fig. 1). The 2021 WHO CNS tumor classification was applied which implied that non-adult patients as well as samples with an isocitrate dehydrogenase mutation (IDHmt) were not eligible [2]. eLTS patients were identified from this cohort, defined as GBM patients who were alive 10 years after diagnosis or 5 years after first recurrence. Samples from which no formalin-fixed, paraffin-embedded (FFPE) material was available, and thus no DNA methylation could be performed, were excluded. From the remaining patient pool, median-term survivors (MTS) were also selected, defined as GBM patients with a survival of 24 months or less. Patients who were still alive with a shorter survival than that of an eLTS were not eligible. Studies were conducted according to ethical guidelines (Declaration of Helsinki). The project was approved by the Ethics Committee of the UHL.

### DNA extraction and DNA-methylation data processing

Twelve fresh, consecutive 5 µm-thick slides were cut from each GBM FFPE sample. The two flanking slides were stained with hematoxylin & eosinophil (H&E) staining. On these flanking H&Es, the histopathological GBM diagnosis was reaffirmed, and the tumor-rich region was indicated based on the notes of a senior neuropathologist (RS). On the ten intermediate unstained slides, DNA was extracted (Maxwell® RSC FFPE Plus DNA kit (AS1720)) from the corresponding regions. One sample was excluded due to doubt regarding the histopathological diagnosis of GBM and was considered a lower grade glioma. Three more samples were excluded after IDH1 immunohistochemical staining or DNA sequencing for IDH1 or IDH2 mutations (*n* = 2 and *n* = 1, respectively). A total of 53 resections from 53 patients were sampled.

DNA concentrations were quantified using the Qubit fluorometer with DNA High sensitivity assay kit. A minimum value of 300 ng of extracted DNA was set to continue methylation analysis. The probability of usable data at lower DNA yield was considered negligible. When possible, a total of 500 ng DNA per sample was preferred. This was subsequently subjected to bisulfite conversion using the EZ DNA Methylation™ kit (50 reactions) (D5001, Zymo research). Illumina Infinium HD FFPE DNA Restore Kit and Zymo Research Clean & concentrator-5 (Capped) (50 preps) (D4013) were used to restore degraded FFPE material and to test and to improve the quality of obtained DNA. Bisulfite-converted DNA was profiled using the Infinium® HumanMethylation EPIC BeadChip (Illumina Inc., San Diego, CA), which examines 865,859 CpG sites. Infinium Methylation EPIC BeadChip Kits(20042130) were used. Scanning was performed using the iScan™ System – Illumina. All of these analyses followed all mandatory laboratory health and safety procedures.

Data were processed using R statistical software version 4.0.2, the LICMEpigenetics package (version 0.1.0), and the ilm10b4.hg19 annotation file, using human genome 19 (hg19) as a reference, which allowed us to identify the exact gene region where the methylation differences occurred [3, 4]. As described later, this package includes R functions for excluding low-quality samples and probes, normalizing methylation data and identifying differentially methylated positions and regions.

The quality of the methylation data was reviewed at both sample level and probe level and corrected where necessary.

First, clear sample outliers of the classical methylation value distribution on the sample histogram (density plot) were excised from further analysis (2 eLTS and 1 MTS) (Supplementary Fig. 1). Beta values (between 0 and 1; respectively referring to no and complete methylation of a methylation site in all the cells of that sample) as well as the corresponding M values (log2 ratio of the intensity of methylated vs. unmethylated probe) were obtained from the raw intensities after background subtraction, color correction, and functional normalization. Background, defined by negative control probes, subtraction was performed by eliminating probes that could not be detected with greater certainty than *p* < 0.01 in more than 50% of the samples (*n* = 7708; [3, 5, 6]).

Probes located on sex chromosomes (*n* = 19,230) and single nucleotide polymorphisms (SNPs) (*n* = 29,721) were also removed from further analysis. No adjustment was made for batch effect given the low number of samples from this unique eLTS group. Samples from both survival groups were run across several batches.

It was then determined which genes corresponded to the differentially methylated regions. For this purpose, hg19-based annotation within the minifi package was used to map DMPs and DMRs on the genome.

First, all CpG sites were examined to identify the differentially methylated positions (DMP) between the group of eLTS patients and the group of MTS patients. This was done by calculating the absolute difference between the beta values for each differentially methylated CpG site per survival group. These were then displayed using the mean and standard deviation. Next, differentially methylated regions (DMR), being DNA regions where a whole groups of CpG sites are on average differentially methylated, were determined via the DMRcate package [7].

Gene Ontology (GO) and Reactome functional annotation analysis was used to identify the main biological processes associated with both differentially methylated/hyper and—hypomethylated genes [8]. For validation, we relied on an external GBM dataset in which histological information, IDH status, survival time and methylation data were downloaded from the Cancer Genome Atlas Database (TCGA) (http://www.cbioportal.org) [9]. We chose the Glioblastoma TCGA Cell 2013 cohort as our data source, which contained all of the former mentioned information.

IDAT files from all patients were uploaded to the DNA methylation-based brain tumour classifier (v12.5; molecularneuropathology.org) for epigenetic classification, and determination of MGMT promoter methylation. A minimum calibrated diagnostic Classifier score of 0.3 was used before assigning an epigenetic diagnosis, which the authors themselves describe as acceptable in a study setting with a low tumour cell count (and given the age of the tissue samples) [10]. Samples with a score below this threshold were considered not epigenetically classifiable. Using this Classifier, the Copy Number Variations (CNV) for each sample were also obtained.

### Statistics

Clinical characteristics were compared using chi-square or fisher exact tests for categorical variables, Mann–Whitney U test for continuous non-normally distributed variables and unpaired Student's t test for normally distributed continuous variables. Differentially methylated CpG positions (DMPs) and regions (DMR) were compared between the two survival groups. The limma framework was used to examine the link between these differently methylated positions and outcome using a linear model, with adjustments for age and gender. Correction for multiple testing was performed using a false discovery rate (FDR) of 0.05 according to Benjamini-Hochberg [11]. Two sample poison rate test was used to calculate the relative difference in the number of CNVs between the two groups (as a proxy for chromosomal instability). The occurrence of a given CNV between the two groups was compared using Fisher's Exact Test.

## Results

GBM samples from 53 patients were collected, consisting of 12 eLTS patients and 41 MTS patients, for DNA methylation analysis. The median overall survival of eLTS patients was 165 months, while that of MTS patients was 14. Age distribution was about the same in both groups at diagnosis (median age was 52 in the eLTS group and 55 in the MTS group) and did not differ significantly in gender (male gender in 58% of the eLTS and 54% of the MTS group) No significant differences in epigenetic GBM subclass—receptor tyrosine kinase (RTK) I, RTK II and mesenchymal (MES)—or in O6-methylguaninemethyltransferase (MGMT) promoter methylation status were found (Table 1, Supplementary Table 1). Three eLTS and 4 MTS samples were epigenetically labeled different from 'GBM'. These were diffuse hemispheric glioma H3 G34-mutant, astrocytoma, IDH-mutant; and high-grade, pleomorphic xanthoastrocytoma. However, in all cases, upon further immunohistochemical and mutational analysis (sanger sequencing; targeted next generation sequencing, NGS) there was no presence of a respective H3 G34, IDH and BRAF mutation, arguing against the alternative epigenetic diagnosis (Table 1). No significant differences in genomic instability (Z =  − 1.14 *p* = 0.25), nor in the occurrence of EGFR amplification (*p* = 0.24), CDKN2A/B deletion (*P* = 0.28) or PTEN deletion (*p* = 0.42) was found (Supplementary Table 4).

**Table 1:** Demographics, clinical and epigenetic characteristics of included GBM patients

Characteristic	eLTS patients (*n* = 12)	MTS patients (*n* = 41)	*p*—value
Age at diagnosis			
Median (IQR) (years)	52 (45–59)	55 (47–59)	0.58
Gender			
Male – no (%)	7 (58)	22 (54)	0.78
Female – no (%)	5 (42)	19 (46)	
Overall survival (OS)			
Median (IQR) (months)	165 (114–183)	14 (10–16)	< 0.01
Primary/secondary GBM			
Primary – no (%)	12 (100)	38 (93)	1
Secondary – no (%)	0	3 (7)	
Treatment^*a*^			
Complete Stupp – no (%)	11 (92)	35 (85)	0.57
Incomplete Stupp – no (%)	1 (8)	6 (15)	
MGMT status			
Methylated – no (%)	6 (50)	12 (29)	0.30
Unmethylated – no (%)	6 (50)	29 (71)	
Epigenetic classification			
GBM – Mes – no (%)	1 (8)	11 (27)	0.26
GBM – RTK1 – no (%)	0	7 (17)	0.32
GBM – RTK2 – no (%)	2 (17)	11 (27)	0.71
Other diagnosis^*b*^ – no (%)	3 (25)	4 (10)	0.18
N.A.^*c*^ – no (%)	6 (50)	8 (20)	0.06

^*a*^Whether or not the patients were treated as initial treatment according to the Stupp protocol or through other modalities (e.g., monotherapy chemotherapy or radiotherapy) [1]

^*b*^Other DNA-methylation based diagnosis included diffuse hemispheric glioma H3 G34-mutant, astrocytoma, IDH-mutant;high-grade, pleomorphic xanthoastrocytoma. However, there was the absence of an H3, IDH and BRAF mutation, respectively, upon further testing via next generation sequencing. Moreover, all samples were anatomopathologically compatible with the diagnosis of GBM

^*c*^*NA* Not assignable. Calibrated score < 0.3

*Mes* Mesenchymal

Three patients (2 eLTS and 1 MTS patient) were subsequently excluded, because the distribution of the normalized CpG methylation pattern showed marked outliers (Supplementary Fig. 1). Sixty-seven DMPs and 5 DMRs were obtained after strict quality correction. An overview of all differentially methylated CpG sites, their corresponding DMP, as well as the DMR, are summarized in Supplementary Table 2. The median of the absolute differences in the mean beta values per DMPs between the two survival groups was 23% (IQR = 13%). The logFC averaged -1.97 (SD 1.22).

**Fig. 2:**
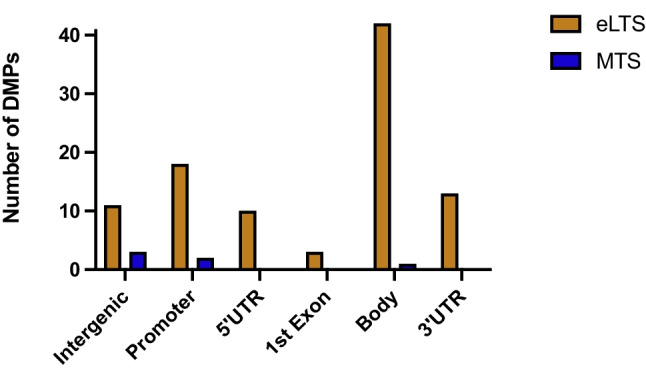
Bar chart depicting the distribution of the Differentially Methylated Positions (DMPs)

Fifty-three of the 67 DMPs were located in 42 unique genes, of which about 38% sites are distributed in the promoter proximal regions (TSS1500, TSS200 and 5′UTR), and the remaining 55% in the gene body and first exons (Fig. 2). Approximately 8% of DMPs were found in 3′ UTR. Five DMPs spanned multiple regions. In addition, all but 5 DMPs, of which 2 intragenic located, were hypermethylated in the eLTS group relative to the MTS group. Five DMRs were identified, and all were globally hypermethylated in eLTS with a median of 11 (IQR 7) CpG sites and 714 base pairs (IQR 297). A complete list of all CpG sites by DMR is shown in Supplementary Table 2. For 4 of the 5 DMRs, the majority of differentially methylated CpG regions were located in genes, with 2 DMRs almost exclusively in the promoter regions, one exclusively in the body and one spanning mixed genetic regions.

When annotating the 53 DMPs containing a gene region according to the Gene Ontology (GO) database, the 3 main biological processes within which they could be placed were "negative regulation of interferon-beta production," "cell adhesion" and "synapse organization.

The most significant, well ahead of the second, differentially methylated pathway using the Reactome pathway database was 'MAPK family signaling cascade'.

When we looked only at the genes where methylation differences were in the promoter region and which were all hypermethylated in eLTS, we saw differences mainly in the pathway 'signaling by NTRK3 (TRKC)', followed by 'Receptor-Type Tyrosine-Protein Phosphatases' and 'Synaptic Adhesion-Like Molecules'. Given only the gene SOCS5 was more hypomethylated in its promoter region in eLTS samples compared to MTS samples, such an analysis could not be performed here.

From the total of 585 patients from the TCGA dataset, there were 365 IDH wild type. Of this latter group, two GBMs met our definition of eLTS and 307 met our MTS definition (Supplementary Table 3). Only one methylated position examined from the UHL group had also been examined in the TCGA database (Supplementary Database 3). (This was not significantly different between the two TCGA survival groups.). In the TCGA database, we identified 1172 (non-preprocessed) significantly different methylation sites between the two survival groups. Analogous to the UHL cohort, the Reactome pathway database was searched for the significantly most differentially methylated pathways. These included 'Signaling by Receptor Tyrosine Kinases', 'MAP1K/MAPK3 signaling', 'RAF/MAP kinase cascade', 'MAPK family signaling cascades', 'Neutrophil degranulation' as well as six other mostly more general pathways (Supplementary Table 3). Since the TCGA database did not further specify in which genetic region the methylation was located, a separate analysis of methylation differences in the promoter region could not be performed.

## Discussion

In this study, methylation differences in CpG sites between samples from the extremely rare group of eLTS patients were compared with those from MTS patients. The extreme long survival as defined for eLTS samples ensures that the survival differences cannot be explained solely by an exhaustive set of known clinical factors, which are known to explain an increase in survival from 3 to 6 months [12–15], but rather due (in part) to the discovered instrinsic variation in methylation patterns. After careful preprocessing, only those DMPs for which we could state with high certainty that they differed between the two groups were retained, despite the old samples and therefore sometimes poorer FFPE quality. Overall, we observed a hypermethylation pattern in the eLTS patients, congruent with reduced interferon beta signaling, which previous studies have shown to be associated with a more fragile glioma stem cell (GSC) population [16]. These GSC are present to a higher degree in MTS and are often seen as a therapy-resistant reservoir from which relapse occurs. Furthermore, differences in methylation of the MAPK signaling pathway seemed particularly important to achieve exceptionally long survival. This was further confirmed by the differently methylated pathways found in eLTS and MTS samples from the CTGA cohort. Given the intrinsic GBM heterogeneity, the lack of preprocessing data in the TCGA database, and the even smaller sample size, an identical result between the UHL and TCGA cohorts is not to be expected, yet 3 of the 11 significantly differentially methylated pathways from the TCGA dataset were related to MAPK signaling. If we looked, in the UHL cohort, only at genes whose promoter region was hypermethylated in eLTS samples, resulting in suppression of gene expression, we saw notably less NTRK3 signaling. Although NTRK abnormalities are extremely rare in glioblastoma, inhibition of these receptors, if present, appears to yield extremely favorable preliminary results in the treatment of this subgroup of glioblastoma [17–19]. These results hint at a select subset of GBMs in which spontaneous, methylation-associated, suppression of NTRK may occur with subsequent favorable survival. Thus, further exploration of this pathway, and thus not just a subset of receptors, seems valuable in discovering new therapeutic options. The absence of MGMT-associated methylation differences between the two survival groups could raise questions. However, although MGMT methylation plays a predictive role in GBM, its value is no longer significant after 5 years of survival [20]. This was also reconfirmed by our data.

Limitations of this study include the relatively small sample size, which may predispose to type 2 errors. However, given the extreme rarity of eLTS samples and the precision with which the diagnosis of GBM was reconfirmed according to WHO 2021 criteria, this is to our knowledge the largest comparable subgroup published to date. Moreover, by defining large survival differences between the two groups, as well as implementing pathway analyses, we tried to limit type 2 errors. Another point to note is the sometimes alternative epigenetic label given to the histologically reconfirmed GBMs. Here it is important to note that this Classifier tool is not yet recognized as a clinically graded tool and is still under development, as highlighted in the latest WHO CNS Classification update [21]. Here, this epigenetic classification served as a readout of a potential explanatory survival difference; given that the eLTS group described here is a unique cohort of GBM patients, it is not illogical that it falls into an epigenetically distinct subgroup of the histologically more homogeneous GBMs, which therefore, in addition to potentially older material quality, results in discrepant epigenetic labeling. Another limitation is the quality of the samples, which is inherent to the sample age associated with studying extremely long survivors. We therefore tried in multiple ways to separate underlying noise from the true signal to detect only the most robust methylation differences. Given the limited material available, further genetic analyses could not take place meaning that the differences found only serve as hypothetical targets and provide a foundation for subsequent confirmatory studies. Finally, regarding the TCGA dataset, it contained only 2 eLTS samples, and an only limited overlapping methylation set without in-depth preprocessing. Therefore, statements about individual DMPs did not seem possible. We thus looked at differently methylated pathways, which allowed us to narrow the dimensionality of our data and highlight significant patterns that individual mutations would miss. However, despite the fact that differences in methylation may explain only part of the overall heterogeneity that predisposes to longer survival, finding similar pathways in both datasets underscores the importance of our findings.

In conclusion, DNA methylation differences are already present at baseline in treatment naive GBM samples that are part of genes and pathways that play a role in survival in these types of tumors. These differences do not coincide in any form with differences in classical epigenetic subtypes of GBM as classified by the Heidelberg classifier (RTK I, RTK II, MES). Since these methylation differences may explain part of the puzzle, and not the entire heterogeneous spectrum that predisposes to long survival in GBM, it is not our intention to present these findings as a comprehensive stand-alone prognostic tool translatable to the individual GBM patient. Rather we want to emphasize the valuable fundamental insights they provide regarding survival. Moreover, these findings appear to be promising targets for future modular antitumor therapies or may serve to assess individual patient sensitivity to future targeted therapies (analogous to PD-L1 in melanomas). Further research will have to determine the exact biological role of the differences found.

### Supplementary Information

Below is the link to the electronic supplementary material.

Supplementary file1 (PDF 536 KB)

Supplementary file2 (XLSX 14 KB)

Supplementary file3 (XLSX 25 KB)

Supplementary file4 (XLSX 104 KB)

Supplementary file5 (XLSX 11 KB)

## Data Availability

No datasets were generated or analysed during the current study.
