# Supplementary figures and images for: A novel methylation signature predicts extreme long-term survival in glioblastoma
Source: J Neurooncol. 2024 Jun 19;169(2):341–7. doi: 10.1007/s11060-024-04741-z (PMC11341684; doi:10.1007/s11060-024-04741-z)

# Excluded

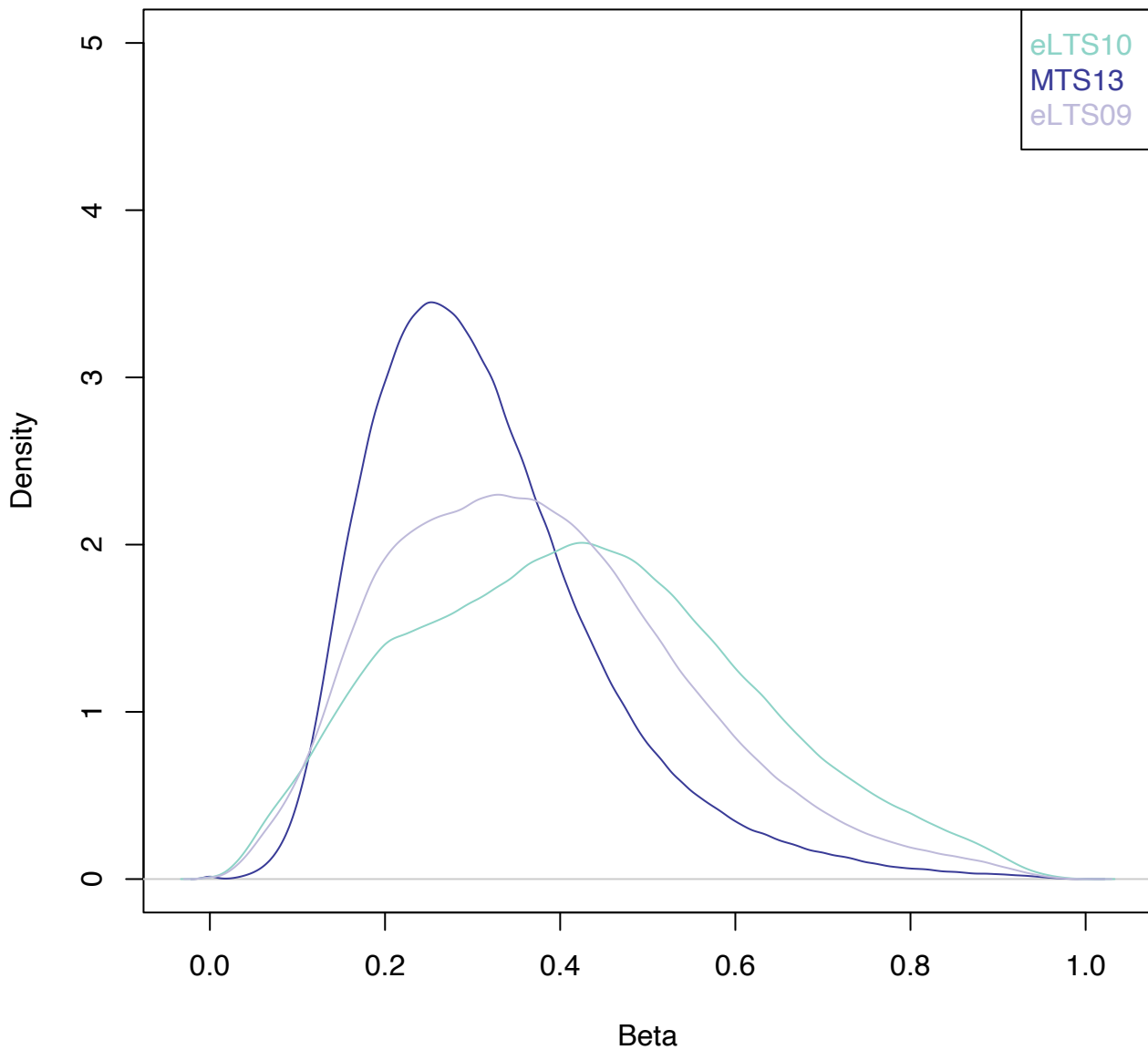

# Included

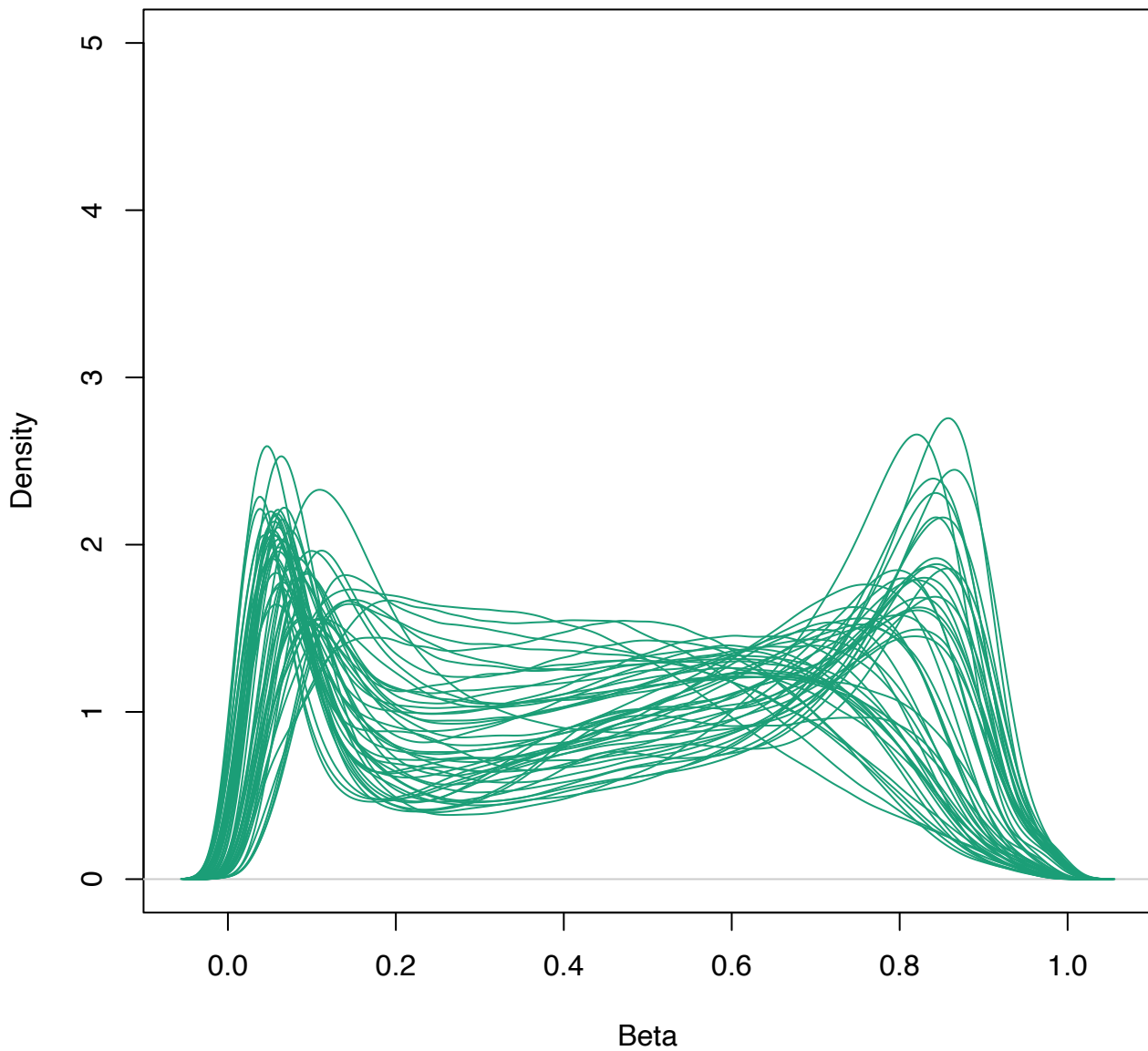

Supplement: Supplementary file 1 — Supplementary file1 (PDF 536 KB) [file 11060_2024_4741_MOESM1_ESM.pdf]
